# Triple Non-Statin Therapy with Ezetimibe, Inclisiran, and Bempedoic Acid in Patients with Genetically Confirmed Statin-Induced Rhabdomyolysis: A Dual Case Report

**DOI:** 10.3390/ph18060818

**Published:** 2025-05-29

**Authors:** Jozef Dodulík, Jiří Plášek, Ivana Kacířová, Romana Uřinovská, Jiří Vrtal, Jan Václavík

**Affiliations:** 1 Department of Internal Medicine and Cardiology, University Hospital Ostrava, 708 00 Ostrava, Czech Republic; jiri.plasek@fno.cz (J.P.); jiri.vrtal@fno.cz (J.V.); jan.vaclavik@fno.cz (J.V.); 2 Centre for Research on Internal and Cardiovascular Diseases, Faculty of Medicine, University of Ostrava, 703 00 Ostrava, Czech Republic; 3 Department of Clinical Pharmacology, Institute of Laboratory Medicine, University Hospital Ostrava, 708 00 Ostrava, Czech Republic; ivana.kacirova@fno.cz (I.K.); romana.urinovska@fno.cz (R.U.); 4 Department of Clinical Pharmacology, Faculty of Medicine, University of Ostrava, 703 00 Ostrava, Czech Republic

**Keywords:** statin intolerance, rhabdomyolysis, inclisiran, bempedoic acid, ezetimibe, genetic testing, dyslipidemia, SLCO1B1

## Abstract

**Background:** Statin intolerance is a serious therapeutic dilemma in secondary cardiovascular prevention (e.g., ESC/EAS Guidelines 2023). This is especially true when confirmed by genetic predisposition and complicated by rhabdomyolysis. Although several non-statin agents have become available in recent years, evidence regarding their combined use in high-risk statin-intolerant patients remains limited. Furthermore, the pharmacokinetics of statins in toxic concentrations are poorly characterized in clinical settings. **Case Presentation:** We present two cases of genetically confirmed statin-induced rhabdomyolysis, both accompanied by severe acute kidney injury requiring renal replacement therapy. In both patients, serial measurements of rosuvastatin plasma concentrations revealed markedly delayed elimination, with detectable levels persisting for several weeks despite ongoing dialysis. Estimated half-lives exceeded 7 days in both cases, far beyond the known therapeutic range. Genetic testing identified *SLCO1B1*, *ABCB1*, and *CYP2C9* polymorphisms linked to reduced hepatic uptake and impaired drug clearance. Following biochemical recovery, both patients were initiated on a triple non-statin lipid-lowering regimen consisting of ezetimibe, bempedoic acid, and inclisiran. The combination was well tolerated, with no recurrence of muscle-related symptoms or biochemical toxicity. LDL-C levels were reduced from 3.05 to 1.59 mmol/L and from 4.99 to 1.52 mmol/L, respectively, with sustained response over 12 and 40 weeks. Full lipid profiles demonstrated favorable changes across all parameters. **Conclusions:** These two cases suggest that the combination of ezetimibe, inclisiran, and bempedoic acid may serve as a safe and effective therapeutic option in patients with severe statin intolerance. Pharmacogenetic testing and serial pharmacokinetic assessment may guide personalized lipid-lowering strategies and improve outcomes in this challenging patient population.

## 1. Introduction

Statins are primarily used to treat dyslipidemia and prevent cardiovascular disease. These drugs reduce levels of low-density lipoprotein cholesterol (LDL-C), a major contributor to atherosclerotic plaque formation in arteries. Statins can reduce LDL-C by up to 55%, as shown in multiple large-scale meta-analyses [1,2], making them among the most effective tools for dyslipidemia treatment and prevention. Statins are currently the first-choice treatment for dyslipidemia according to the latest (and previous) European Society of Cardiology (ESC) guidelines, based on class I recommendations (level of evidence A) [3]. The most common adverse effects of statins include myalgia (1–10%), various non-specific gastrointestinal complaints (<5%), elevated liver function markers (0.5–2%), and increased risk of diabetes (0.1–0.2%) [4,5], though estimates vary among populations. Other adverse effects are rare.

True statin intolerance is reported in approximately 5–10% of patients, though confirmed rhabdomyolysis is rare (<0.1%) [5]. In cases of statin intolerance or serious adverse effects, such as rhabdomyolysis, alternative lipid-lowering therapies are available, including ezetimibe, proprotein convertase subtilisin/kexin type 9 inhibitors (PCSK9i), bempedoic acid, inclisiran, and bile acid absorbers [3]. Some combinations of these hypolipidemics have been investigated in clinical trials, but limited data exist for newer combinations such as bempedoic acid and incliciran [6,7,8].

Here, we present two cases of genetically confirmed statin-induced rhabdomyolysis successfully managed with a combination of ezetimibe, inclisiran, and bempedoic acid. These cases illustrate the clinical potential of a triple non-statin lipid-lowering regimen in patients at high cardiovascular risk who are unable to tolerate statin therapy.

## 2. Case Presentation

### 2.1. Patient 1

A 56-year-old woman with a history of premature atherosclerotic cardiovascular disease (ASCVD), arterial hypertension, and active smoking was admitted to the coronary care unit with an acute anterior ST-elevation myocardial infarction (STEMI). Coronary angiography revealed complete occlusion of the proximal left anterior descending (LAD), which was successfully revascularized. High-intensity lipid-lowering therapy with rosuvastatin 40 mg/day was initiated on the second day after STEMI.

**Figure 1:**
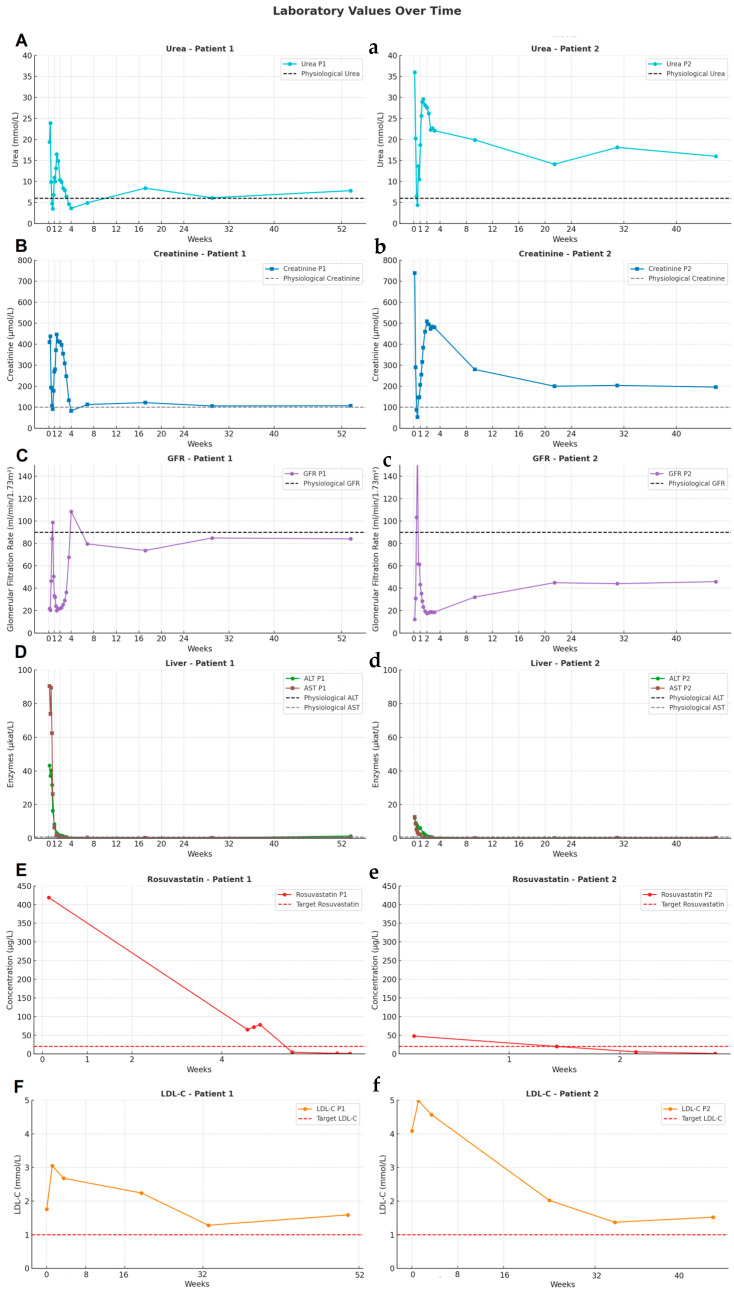
Laboratory Values Over Time. (**A**,**a**) Urea values, (**B**,**b**) Creatinine values, (**C**,**c**) Glomerular filtration rate (GFR) values, (**D**,**d**) Liver enzyme values, (**E**,**e**) Rosuvastatin plasma concentrations over time, (**F**,**f**) LDL-C values.

During hospitalization, the patient developed progressive myalgia and muscle weakness. Laboratory evaluation revealed marked elevations (Figure 1A shows urea levels, Figure 1B creatinine, Figure 1C glomerular filtration, and Figure 1D liver enzymes) in creatine kinase (CK 23,260 U/L), myoglobin (>3000 µg/L), liver enzymes (AST 90.4 µkat/L, ALT 43.2 µkat/L), and acute kidney injury (AKI) (creatinine peak 446 µmol/L, urea 16.5 mmol/L). Due to worsening renal function, hemodialysis was initiated. Rosuvastatin was discontinued.

Given the severity of the clinical presentation, serial plasma rosuvastatin concentrations were monitored, revealing extreme and prolonged systemic exposure. On day 1, the concentration was 418.7 µg/L (reference therapeutic range: 5–20 µg/L). The decline was unexpectedly slow, with complete elimination occurring after 48 days, despite repeated haemodialysis during hospitalization and supportive care. No additional drug interactions were identified that could have contributed to prolonged elimination. The full kinetic profile is shown in Figure 1E.

Pharmacogenetic testing revealed heterozygosity for the *SLCO1B1* c.521T > C polymorphism and *ABCB1* gene variants, both linked to impaired hepatic uptake and biliary excretion of statins. These factors likely contributed to the toxic accumulation and prolonged elimination of rosuvastatin.

Following normalization of CK, liver enzymes, and renal function (Figure 1A–D), the patient was initiated on non-statin lipid-lowering therapy consisting of ezetimibe 10 mg/day, bempedoic acid 180 mg/day, and inclisiran 284 mg subcutaneously every six months. Ezetimib was reintroduced during hospitalization (day 12). Bempedoic acid was started three weeks after discharge (day 50), and inclisiran was administered 10 weeks later (day 99). The regimen was well tolerated, and LDL-C was reduced from 3.05 to 1.59 mmol/L (Figure 1F). Full lipid profile changes are presented in Table 1A,B. No further adverse events occurred during the 12-month follow-up.

### 2.2. Patient 2

A 64-year-old woman with a history of arterial hypertension, ASCVD, and prior coronary artery bypass grafting (CABG) was referred to our clinic for progressive fatigue and laboratory signs of rhabdomyolysis. Several months prior, she had experienced myalgia, weakness, and elevated CK following rosuvastatin therapy, which resolved upon discontinuation.

At the time of referral, the patient was asymptomatic and without statin therapy. Due to her cardiovascular history, rosuvastatin 40 mg/day was reinitiated. The patient developed muscle pain and increasing fatigue on day 3 of rosuvastatin therapy. Laboratory testing revealed CK 12,580 U/L, elevated AST (12.6 µkat/L) and ALT (11.9 µkat/L), and AKI with creatinine 739 µmol/L and urea 36 mmol/L (Figure 1(a–d)).

Rosuvastatin was immediately discontinued, and supportive care was initiated. Given the prior statin-associated episode and current severity, plasma rosuvastatin levels were monitored, from 47.7 µg/L on day 1 to 1.0 µg/L on day 20 (reference range: 0.1–2.0 µg/L), again confirming prolonged systemic exposure beyond the expected elimination timeframe. Values and kinetics are visualized in Figure 1(e).

Pharmacogenetic testing revealed the same *SLCO1B1* c.521T > C polymorphism as in Patient 1, along with a relevant *CYP2C9* variant. These genetic findings further supported a pharmacokinetic basis for statin intolerance with impaired elimination and increased toxicity risk.

Once renal function stabilized and laboratory parameters normalized, a non-statin triple regimen was initiated: ezetimibe 10 mg/day, bempedoic acid 180 mg/day, and inclisiran 284 mg subcutaneously every 6 months. Ezetimibe was reintroduced on day 27. Inclisiran was administered as a single subcutaneous injection on day 62, and bempedoic acid was initiated on day 146, twelve weeks after inclisiran. The patient tolerated the combination well and remained asymptomatic.

At 40 weeks of follow-up, LDL-C had decreased from 4.99 to 1.52 mmol/L, as shown in Figure 1(f). Comprehensive lipid data are summarized in Table 2A,B. No recurrence of muscle symptoms or organ dysfunction occurred.

## 3. Discussion

Statins are the gold standard for LDL-C reduction and cardiovascular risk mitigation; however, true statin intolerance affects approximately 5–10% of patients, though genetically confirmed statin-induced rhabdomyolysis remains rare (<0.1%) [9]. Our cases illustrate that in genetically predisposed individuals, standard statin therapy can result in life-threatening adverse effects, necessitating alternative therapeutic strategies.

A unique aspect of our report is the detailed analysis of rosuvastatin plasma kinetics during the acute phase of rhabdomyolysis. Both patients demonstrated markedly elevated and prolonged rosuvastatin concentrations, with estimated elimination half-lives of 7.5 days, compared to the typical 19 h reported in healthy individuals [10]. Importantly, this delay persisted despite renal replacement therapy, suggesting that hepatic uptake and biliary excretion were the primary limiting factors. The presence of *SLCO1B1 c.521T > C* polymorphism and variants in *ABCB1* and *CYP2C9* genes, detected in both cases, supports the hypothesis of impaired hepatic clearance contributing to systemic drug accumulation and toxicity [2,11].

Although non-statin therapies such as ezetimibe, PCSK9i, bempedoic acid, and inclisiran are individually approved for LDL-C lowering [12,13,14,15], there is limited evidence regarding their combined use in patients with confirmed statin intolerance and prior rhabdomyolysis. A recent network meta-analysis supports the superior LDL-C lowering efficacy of inclisiran over ezetimibe and bempedoic acid, reinforcing its role in combination regimens [16]. To our knowledge, no published trials to date have systematically assessed the triple combination of ezetimibe, inclisiran, and bempedoic acid in this specific population. Our approach utilizing triple non-statin therapy with ezetimibe, inclisiran, and bempedoic acid achieved sustained LDL-C reductions in both patients, without recurrence of muscle-related symptoms or biochemical abnormalities during a 12-month follow up for Patient 1 and a 40-week follow-up for Patient 2.

The choice of inclisiran instead of monoclonal antibody PCSK9i was based on several considerations. Inclisiran, a small interfering RNA-targeting hepatic PCSK9 production, requires administration only twice a year after the initial two doses, offering a significant advantage in terms of patient adherence and healthcare resource optimization [12,15]. Its overall safety and tolerability profiles have been well established in recent reviews [17]. In addition, in many healthcare systems, inclisiran is more accessible and economically favorable compared to PCSK9 monoclonal antibodies [15]. However, access and reimbursement policies for inclisiran vary widely between countries, potentially limiting its availability in some healthcare settings [15].

Our cases also highlight the emerging role of genetic testing in the management of dyslipidemia. Identification of pharmacogenetic variants associated with statin intolerance, such as *SLCO1B1* polymorphisms, may aid in early diagnosis and guide the choice of lipid-lowering therapies, preventing unnecessary adverse events and treatment discontinuation [18]. Nevertheless, the clinical utility of routine genetic screening remains controversial due to costs and limited availability in many regions.

To our knowledge, this is the first report documenting the practical feasibility, tolerability, and effectiveness of a triple non-statin regimen based on ezetimibe, inclisiran, and bempedoic acid in patients with genetically confirmed statin-induced rhabdomyolysis. Although limited by the number of cases, our experience suggests that such an approach can successfully achieve lipid targets in this difficult-to-treat population.

As of May 2025, which corresponds to an additional 7 months of follow-up after the previously reported results, both patients remain on the same triple non-statin therapy, with no recurrence of muscle-related symptoms or adverse events. In Patient 1, LDL-C levels have remained stable between 1.37–1.82 mmol/L, urea levels around 16 mmol/L, and creatinine levels around 200 µmol/L. In Patient 2, LDL-C levels have remained stable between 1.68–2.0 mmol/L, urea around 9 mmol/L, and creatinine around 110 µmol/L. Liver function tests remain within physiological ranges in both cases.

Future larger studies are warranted to validate these findings and to establish standardized treatment algorithms for patients with severe statin intolerance confirmed by genetic testing. Such studies should ideally include multicenter cohorts with long-term follow-up and evaluation of both clinical outcomes and pharmacoeconomic aspects.

Although limited by the small sample size, both cases were followed for extended periods (12 months and 40 weeks, respectively), during which the triple non-statin therapy demonstrated sustained LDL-C reduction and excellent tolerability without recurrence of muscle symptoms. These findings support the potential feasibility of this regimen in clinical practice.

## 4. Conclusions

These two case reports highlight the feasibility, tolerability, and effectiveness of triple non-statin lipid-lowering therapy with ezetimibe, inclisiran, and bempedoic acid in patients with genetically confirmed statin-induced rhabdomyolysis. In both cases, the combination therapy enabled sustained LDL-C reduction to below recommended targets without recurrence of muscle-related adverse effects. Detailed pharmacokinetic monitoring revealed markedly delayed rosuvastatin elimination, likely attributable to genetic polymorphisms affecting hepatic drug handling. Our findings support the role of personalized lipid-lowering strategies, including the use of genetic testing, in optimizing care for patients with severe statin intolerance.

## Figures and Tables

**Table 1:** (**A**) Complete lipid profile before and after non-statin therapy in mmol/L. (**B**) Complete lipid profile before and after non-statin therapy in mg/dL.

	(A) Patient 1		(B) Patient 2	
Parameter	Before (mmol/L)	After (mmol/L)	Before (mmol/L)	After (mmol/L)
TC	4.02	2.63	6.99	3.48
HDL-C	1.04	0.89	1.21	1.6
Non-HDL-C	2.98	1.74	5.78	1.88
LDL-C	3.05	1.59	4.99	1.52
TAG	1.4	1.07	2.57	1.25
APOA1	1.24	1.05	1.04	1.58
APOB	0.86	0.51	1.36	0.57
Lp(a)	0.804	0.859	1.54	1.11

TC = Total Cholesterol; TAG = Triglycerides; APOA1 = Apolipoprotein A1; APOB = Apolipoprotein B; Lp(a) = Lipoprotein(a).

**Table 2:** (**A**) Complete lipid profile before and after non-statin therapy in mmol/L. (**B**) Complete lipid profile before and after non-statin therapy in mg/dL.

	(A) Patient 1		(B) Patient 2	
Parameter	Before (mg/dL)	After (mg/dL)	Before (mg/dL)	After (mg/dL)
TC	155.5	101.7	270.3	134.6
HDL-C	40.2	34.4	46.8	61.9
Non-HDL-C	115.2	67.3	223.5	72.7
LDL-C	124	94.8	227.6	110.7
TAG	48	40.6	40.2	61.1
APOA1	33.3	19.7	52.6	22
APOB	31.1	33.2	59.8	42.9
Lp(a)	117.9	61.5	193	58.8

TC = Total Cholesterol; TAG = Triglycerides; APOA1 = Apolipoprotein A1; APOB = Apolipoprotein B; Lp(a) = Lipoprotein(a).

## Data Availability

The data presented in this study are available on request from the corresponding author. The data are not publicly available due to patient confidentiality, individual patient data are not publicly available.
